# Kirchhoff’s Thermal Radiation from Lithography-Free Black Metals

**DOI:** 10.3390/mi11090824

**Published:** 2020-08-30

**Authors:** Takuhiro Kumagai, Naoki To, Armandas Balčytis, Gediminas Seniutinas, Saulius Juodkazis, Yoshiaki Nishijima

**Affiliations:** 1 Department of Physics, Electrical and Computer Engineering, Graduate School of Engineering, Yokohama National University, 79-5 Tokiwadai, Hodogaya-ku, Yokohama 240-8501, Japan; kumagai-takuhiro-hv@ynu.jp (T.K.); to-naoki-jp@ynu.jp (N.T.); armandas.balcytis@gmail.com (A.B.); 2 Center for Physical Sciences and Technology, A. Goštauto 9, LT-01108 Vilnius, Lithuania; 3 Optical Sciences Centre and ARC Training Centre in Surface Engineering for Advanced Materials (SEAM), School of Science, Swinburne University of Technology, Hawthorn, VIC 3122, Australia; gdseniutinas@gmail.com (G.S.); saulius.juodkazis@gmail.com (S.J.); 4 Institute of Advanced Sciences, Yokohama National University, 79-5 Tokiwadai, Hodogaya-ku, Yokohama 240-8501, Japan; 5 Tokyo Tech World Research Hub Initiative (WRHI), Tokyo Institute of Technology, School of Materials and Chemical Technology, 2-12-1, Ookayama, Meguro-ku, Tokyo 152-8550, Japan

**Keywords:** thermal radiation, metasurface, black metal

## Abstract

Lithography-free black metals composed of a nano-layered stack of materials are attractive not only due to their optical properties but also by virtue of fabrication simplicity and the cost reduction of devices based on such structures. We demonstrate multi-layer black metal layered structures with engineered electromagnetic absorption in the mid-infrared (MIR) wavelength range. Characterization of thin ${\mathrm{SiO}}_{2}$ and Si films sandwiched between two Au layers by way of experimental electromagnetic radiation absorption and thermal radiation emission measurements as well as finite difference time domain (FDTD) numerical simulations is presented. Comparison of experimental and simulation data derived optical properties of multi-layer black metals provide guidelines for absorber/emitter structure design and potential applications. In addition, relatively simple lithography-free multi-layer structures are shown to exhibit absorber/emitter performance that is on par with what is reported in the literature for considerably more elaborate nano/micro-scale patterned metasurfaces.

## 1. Introduction

Metasurface absorbers, composed of patterns of sub-wavelength antennas, are used for tailoring spectral, polarisation and angular dependencies of electromagnetic radiation absorption as well as thermal radiation emission [1,2,3,4,5,6,7,8,9,10,11]. The Kirchhoff’s law of thermal radiation describes the equivalence between absorptivity and emissivity, which is important in applications of thermal radiation and IR light sources for mid infrared (IR) sensors [12,13,14,15,16,17,18,19,20,21,22,23,24]. The radiant output power can easily be tuned by heating of metasurfaces to high values enabled by established commercial light emitting diodes or quantum cascade lasers in mid-IR spectral range. In our previous work, we have demonstrated the feasibility of plasmonic metasurfaces as thermal radiation sources and realized near perfect absorption using R≤ 5% anti-reflective metasurfaces [1,25]. However, nano-structure arrays typical for such applications necessitate the use of high resolution lithography systems, e.g., electron beam lithography (EBL), size reduction photo-lithography based on steppers, or focused ion beam (FIB) nanofabrication [26]. Therefore, a lithography-free approach for fabrication of absorbers has inherent advantages, especially as it eschews the considerable up-front cost of high resolution patterning equipment, that gets incorporated into the end-user price of the fabricated devices, in particular, when macroscopic area coverage has to be achieved [27].

The principle of anti-reflection is necessitated by a desire to increase the efficiency at which electromagnetic energy can be exploited through its interaction with different functional materials. As mater is comprised of charged particles that can be driven by external fields, re-radiation at the material interface, simply called reflectance, is generally expected, and can be especially strong in metals or plasmas due to high densities of quasi-free electrons. Anti-reflection can be described in terms of impedance matching between materials with disparate electromagnatic radiation propagation properties via the effective impedance $Z_{eff}$:

(1) $$Z_{eff}=\sqrt{\frac{\mu_{eff}}{\varepsilon_{eff}}}=1,$$

where $\mu_{eff}$ and $\varepsilon_{eff}$ are the effective permeability and permittivity, respectively. In most cases an equivalent electric circuit model can be used to describe optical nano-/micro-elements forming metasurfaces and films [28]. Due to its inherent generality, the idea of impedance matching holds regardless of the presence or absence of nanostructures as long as the effective medium approximation can be justified.

There are several lithography-free approaches for fabricating structures with light absorption properties. One of the most notable is based on the Tamm plasmon polaritons supported by a thin metal film on a multi-layer Bragg reflector stack [29,30,31,32,33]. Other useful materials include so-called black metals or plasmon super absorbers [33,34,35,36,37], which are comprised of a metal ground plate and a thin overlaying semiconductor film [28,36,38]. Absorber design can be optimized for reduced reflectivity and transmittance using the aforementioned optical impedance matching method [28], which is particularly promising when the refractive index $\sqrt{\varepsilon }\equiv \tilde{n}=n+i\kappa$ (where ε is the permittivity) of the actual thin film is well known.

Some target applications for black metals include the detection of MIR radiation and as thermal emitters in the field of gas sensing [2]. For this purpose, it is important to tune the spectral properties of absorption at specific wavelengths, which in conventional metamaterials is usually done by adjusting the geometry or placement of nanoantennas. Reciprocity between emittance, *E*, and absorbance, *A*, has been confirmed experimentally at the MIR spectral range [2].

Here we experimentally demonstrate MIR black metals composed of two different dielectric layers wedged between metal films, which are capable of electromagnetic absorption and thermoradiative emission in the wavelength region spanning from 2 to 10 μm. These lithography-free multi-layer structures simplify fabrication of black metals that can be used for both spectrally selective thermal radiation emittance and infrared absorbance.

## 2. Experiments

### 2.1. Fabrication of Mid-Infrared (MIR) Black Metals

Multi-layer black metals can be fabricated using conventional physical vapour deposition (PVD) method for thin-film creation. In this study, we have deposited metals and dielectrics on double side mirror polished Si substrates using an electron beam (EB) heating-based evaporator (CANON ANELVA, Kawasaki, Japan). The vacuum chamber was evacuated down to 5.0 × 10^−5^ Pa using a turbo molecular pump (Pfeiffer vacuum. Gmbh, Aßlar, Germany ). Evaporation targets were obtained from commercial suppliers—Si, ${\mathrm{SiO}}_{2}$ and Ti from Kojundo Chemical Laboratory Co. Ltd., (Tokyo, Japan.) Au from Tanaka Kikinzoku Kogyo K.K. (Tokyo, Japan). All the targets had a material purity above 99.99%. First, a foundation 200 nm Au layer was deposited on the Si substrate. Then ${\mathrm{SiO}}_{2}$ (with specific thicknesses in the range between 100 nm and 300 nm) and Si (with a 200–400 nm thickness range) were evaporated. Lastly, the structure was capped by a 15-nm-thick layer of Au. Ultra-thin 3 nm layers of Ti were deposited as adhesion promoting layers between the three metal (Au) and dielectric (Si and ${\mathrm{SiO}}_{2}$) interfaces. Conversely, the intrinsic adhesion between Si and ${\mathrm{SiO}}_{2}$ was strong enough for thermal radiation applications, as it was sufficient to withstand the temperature change induced expansion and contraction stresses. During all PVD steps the material deposition rate has measured using a quartz micro-balance (QCM) and controlled by altering the heating electron beam current.

Optical characterisation of the fabricated metasurfaces was performed using a set of micro-spectroscopic techniques. Reflectance spectra in the wavelength region spanning from visible to near infrared (350 to 1200 nm) were acquired using a measurement setup comprised of a conventional microscope (BX-53, Olympus, Tokyo, Japan) combined with spectrometer (SpectraPro and PXIS, Princeton, NJ, USA). Similarly, reflection measurements in the mid infrared wavelength region employed a conventional Fourier transform-infrared (FT-IR) spectroscope (FTIR 6200, Jasco, Tokyo, Japan) that was combined with an IR microscope unit (IRT-1000, Jasco). Thermal radiation emission was measured with the same FT-IR spectroscope system (FTIR 6200, Jasco) that was rearranged so that the black metal sample was setup as an external IR radiation source. During these thermal radiation emission measurements samples were heated up to a 300 °C temperature. In all cases, ultra broadband dielectric mirror (for reflection of visible to near infrared), Au mirror (for reflection of mid infrared) and 95% efficient black body radiator ink (for thermal radiation emission) were used as spectral references.

### 2.2. Numerical Simulation

Finite difference time domain (FDTD) simulations were performed using a home built workstation based on the AMD Ryzen 7 2700X, 8 core, 16 thread CPU and equipped with 32GB DDR4 memory. A commercially available finite-difference time-domain electromagnetic solver software Lumerical FDTD Solutions was used for simulations. Full 3D simulations of the multi-layer stack were performed. The simulation region has set to span a 3 × 3 μm^2^ region in the xy plane, and extend 10 μm along the *z*-axis. The dielectric parameters of reported by Palik, which cover a broad wavelength range from visible to mid infrared wavelength, were assumed for all metals and dielectric materials. The structure was excited using a broadband pulsed plane wave light source set to propagate along *z*-axis. The simulation region in the *x*- and *y*-axes direction was terminated using a periodic boundary condition, whereas for *z*-axis a perfect matching layer (PML) was chosen so that all radiation is absorbed at the boundary.

## 3. Results and Discussion

### 3.1. Experimental Results

**Figure 1:**
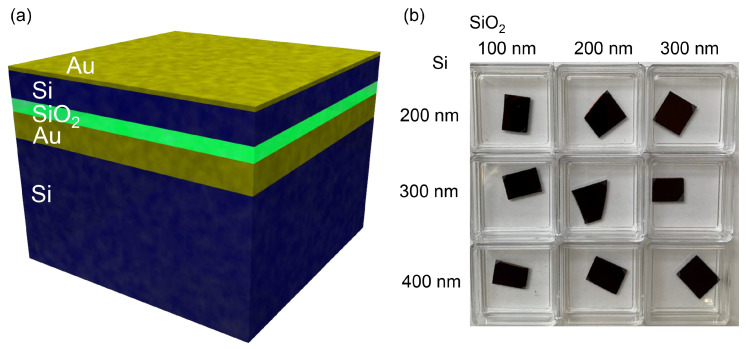
(**a**) Schematic illustration of fabricated multi-layer black metals. (**b**) Photographs of the black metal samples with different semiconductor and dielectric layers.

A schematic sketch of the multi-layered black metals with Si and ${\mathrm{SiO}}_{2}$ layers is depicted in Figure 1. Due to the strong adhesion between Si and ${\mathrm{SiO}}_{2}$ films no additional intermediary layers were required. Conversely, the visualization does not show the aforementioned 3 nm thickness Ti adhesion layers that were deposited to form robust interfaces between Si and Au as well as ${\mathrm{SiO}}_{2}$ and Au. Alternatively, chromium can also be used as an adhesive between Au and dielectrics, however, it is unsuitable for thermal emitter applications, as heating can result in Cr readily diffusing into Au as well as layer de-lamination. Moreover, spectral properties of emitters can be altered by the presence of even a thin layer of Cr, as will be further detailed in Section 3.3. The material evaporation step was carried out over the entire surface of an 8-inch Si wafer and yielded a homogeneous black metal coating, however, for simplified handling during experimental characterization, the Si substrate was cut into smaller pieces. Upon visual inspection fabricated black metals had a dark brown appearance, as illustrated by photographs in Figure 1b.

**Figure 2:**
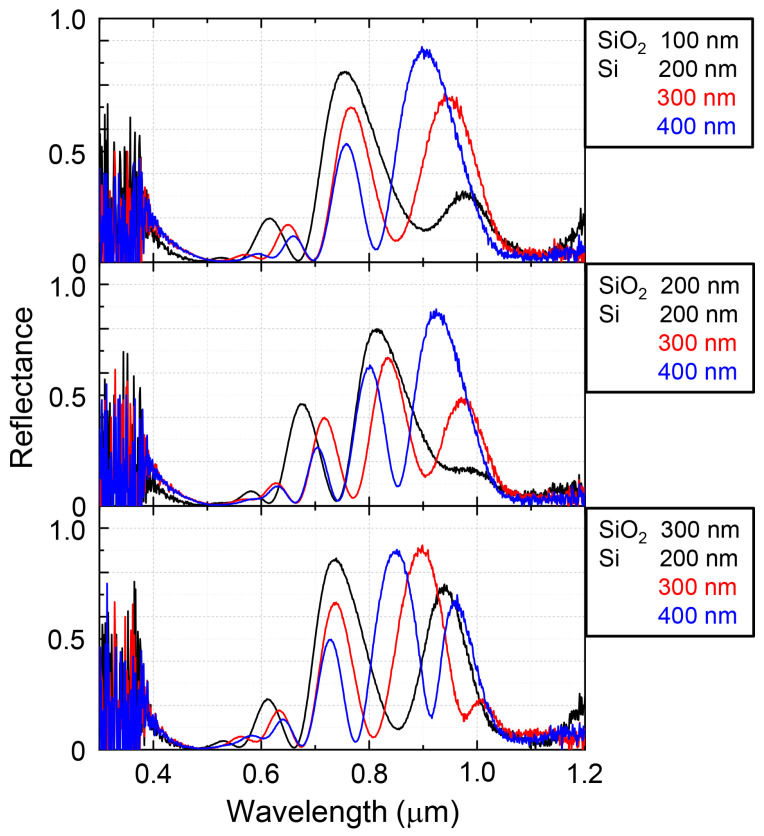
Reflection spectra of black metals from visible to near-IR wavelength range for different thickness of ${\mathrm{SiO}}_{2}$ and Si.

To elucidate the underlying reason for the brown/black color visual appearance of samples, the reflection spectra spanning from visible to near-IR wavelength range were measured. The results are summarized in Figure 2. All samples exhibited a lower than 10% reflectivity in the 400 nm to 600 nm wavelength range. However, at longer wavelengths beyond 600 nm an oscillatory reflection was observed. Throughout the visible to near-IR wavelength region the samples exhibited either two or three alternating absorption and reflectance bands. These peaks, to which the origin of the apparent color of samples is attributed, can be categorized as higher order replicas of mid-IR wavelength absorbance bands. When the Si layer thickness was increased reflectance suppression in the visible wavelength region became larger. This is expected, since silicon is highly absorbing at those wavelengths due to its bandgap of $E_{g}=1.12$ eV, equivalent to λ (μm) $=1.24/(E_{g}$ (eV)) = 1.11 μm.

**Figure 3:**
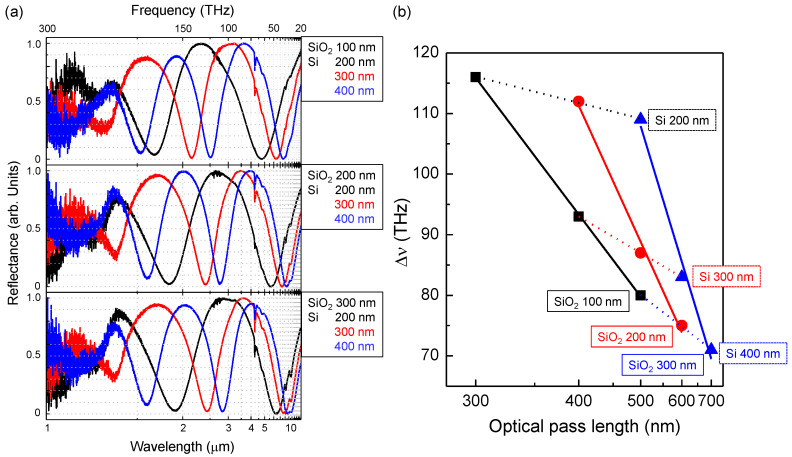
(**a**) Reflection spectra of the fabricated black metal samples with ${\mathrm{SiO}}_{2}$ layer thickness changing from 100 to 300 nm and Si from 200 to 400 nm. (**b**) The full width at half maximum (FWHM) Δν vs. optical path length, calculated as combined thickness of Si and ${\mathrm{SiO}}_{2}$ layers at normal incidence. The slope of the dependence defines the group index $n_{g}$.

Figure 3a shows the complementary experimental reflectance spectra of fabricated black metals over a spectral range spanning from near-to-mid IR (1–10 μm wavelengths). The first order absorbance peaks appeared in the range from 5 to 10 μm. Towards the longer mid-IR wavelengths reflection peaks become broader with full-width at half maximum (FWHM) of Δλ∼5 μm. In the frequency scale, most of the signatures possess comparable widths and uniform peak separations Δν, which are defined by the free spectral range (FSR), defined as:

(2) $$FSR=c/n_{g}L,$$

where *c* is the speed of light, $n_{g}$ is the group index, and *L* is the optical path length. From analysis of FSR (Figure 3b), $n_{g}$ is somewhat wavelength-dependent and falls in the range between 5 and 9. Group index is the indicator of light-matter interaction magnitude, where a higher group index indicates a strong light-matter interaction. For example, a typical $n_{g}$ in silicon waveguide is in the range between 4 and 5, but in a photonic crystal waveguide it can reach up to 60 [39,40]. With a larger $n_{g}$, photonic crystals can be used for stronger linear and non-linear optical interactions over a smaller optical path length. In our case, a large $n_{g}$ would affect the linewidth and absorption manifested in the optical reflection spectra. Thinner ${\mathrm{SiO}}_{2}$ and Si films result in a larger FSR, e.g., ${\mathrm{SiO}}_{2}$ of 100 nm and Si of 200 nm yields in $n_{g}$ = 8.6, whereas 300 nm ${\mathrm{SiO}}_{2}$ and 400 nm Si layer thicknesses correspond to $n_{g}$ = 5.5. Both ${\mathrm{SiO}}_{2}$ and Si thickness were inversely proportional to the total optical path length (Figure 3b). The influence of Si thickness on Δν is much more pronounced than that of ${\mathrm{SiO}}_{2}$ due to the higher refractive index. Experimentally measured absorption at the reflection dips was large and approached nearly the perfect absorption condition (in all cases the transmittance was T=0 due to the fully opaque thick Au bottom layer).

**Figure 4:**
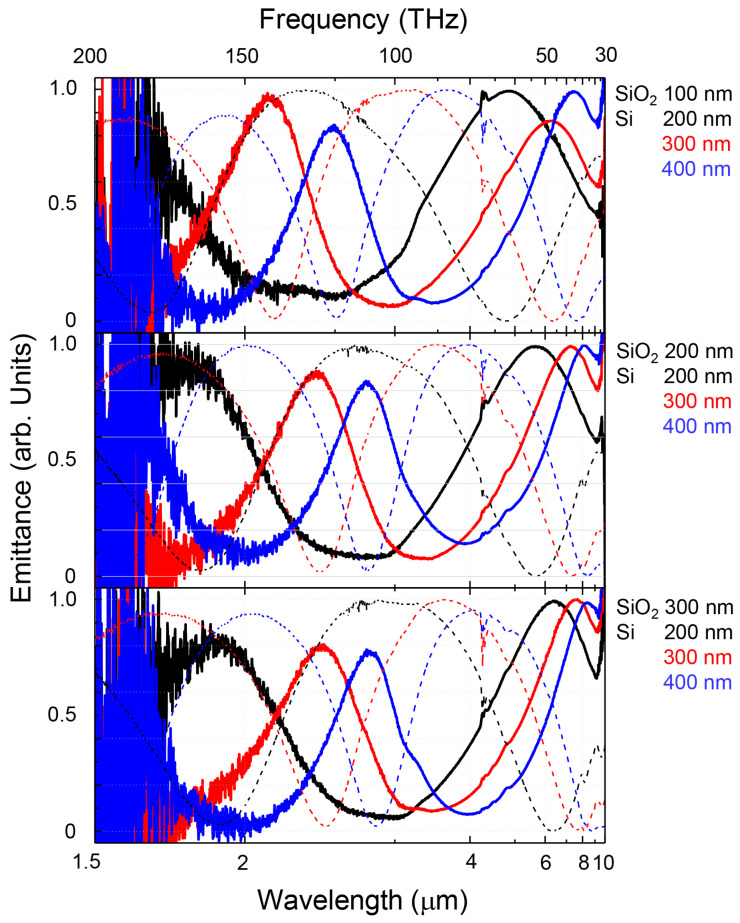
Experimental thermal radiation emission spectra of black metals; dashed lines are the reflectance spectra from Figure 3a. Thermodynamic equivalence based on energy conservation for absorbance, reflectance, and transmittance A+R+T=1 requires that emittance E=1−R=A in the absence of transmittance, as is the case considered here due to a thick Au back reflector layer; Figure 1a.

Figure 4 shows the black body normalized thermal radiation emission spectra of multi-layer black metals heated up to a 300 °C temperature. Due to the limited detection range of the HgCdTe (MCT) bolometer detector installed in the Fourier transform IR (FT-IR) spectrometer used for the measurements, only the spectral range from 1.5 to 10 μm could be reliably probed. In this wavelength region the experimentally obtained emission and reflection spectra were in close accordance to the reciprocity expected from the thermodynamic equivalence between absorbance and emittance E=A. The peak wavelengths of reflectance matched well with emittance dips and vice versa. Emissivity in the longer wavelength range becomes close to 95% of that expected for black body radiation. In most cases the emittance of the second mode, which appeared around 2 to 4 μm became smaller than that of the first mode. In this region, there is no absorption by Si and ${\mathrm{SiO}}_{2}$, however, energy might be lost to the coupling into propagation of plasmon or other lossy lateral modes supported by the planar waveguide formed between the two metal coatings (Figure 1a).

### 3.2. Numerical Simulations

**Figure 5:**
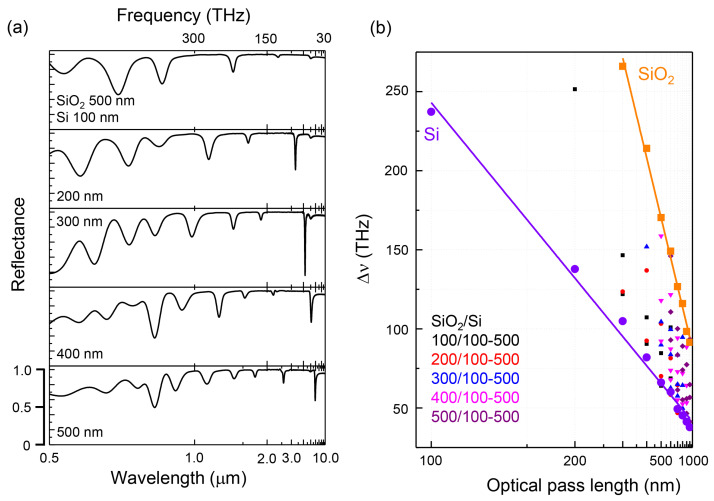
Finite difference time domain (FDTD) simulations (Lumerical FDTD Solutions) of multilayered black metals. (**a**) Reflection spectra of the ${\mathrm{SiO}}_{2}$ 500 nm and a variable Si layer with thickness spanning 100–500 nm. (**b**) The FSR of the ${\mathrm{SiO}}_{2}$ and Si layers with a range of thicknesses from 100 to 500 nm; the bounding lines represent homogeneous Si and ${\mathrm{SiO}}_{2}$ dielectric spacer cases.

Finite difference time domain (FDTD) modeling is well suited to simulate optical properties of black metals as long as the effective medium approximation can be assumed to hold. Interestingly, for the fabricated multi-layer structures, the FDTD results were not conclusive at the expected quantitative level. The FDTD calculation-derived data are summarised in Figure 5. In FDTD simulation, FSR became a complex number. Pure Si and ${\mathrm{SiO}}_{2}$ generate relatively uniform and regularly spaced peaks over the simulated frequency range. All the FSRs should be located between the lines for the pure Si and ${\mathrm{SiO}}_{2}$ (Figure 5b). The complex FSR numbers hints that the multi-reflections had to occur at the boundaries of Au/Si, Si/${\mathrm{SiO}}_{2}$ and ${\mathrm{SiO}}_{2}$/Au. However, the absence of such irregularities in experimental spectra indicate that there could be some intermixing between the Si and ${\mathrm{SiO}}_{2}$ materials and the boudary between them might not be abrupt as abrupt as assumed in simulation.

We used incoherent and un-polarized light in experiment which is different from the FDTD simulations where coherent effects are captured. Moreover, the actual samples are expected to have some roughness on the surfaces and interfaces which otherwise were assumed to be ideal mirror-like in simulations. Therefore the optical properties determined experimentally became broadened and difficult to completely reproduce by FDTD, which used a coherent, polarized light with smooth surfaces. Also, a non-uniform mesh with a small mesh size could be a cause of aliasing in Fourier transform. More detailed study of correspondence between experiment and FDTD simulations were reported in the previous study [1]. It is noteworthy, that FDTD codes for the slow light modes (large $n_{g}$) are specially developed in order to capture absorption and interference in photonic crystal for light trapping in solar cells at the edge of absorption band [41]. Hence, a standard commercial FDTD software is not optimised for modelling slow light absorption.

### 3.3. The Effect of Adhesion Layer

Experimental realization of plasmonic or highly reflective optical structures often relies on noble metals, particularly Ag due to its low losses and Au specifically chosen in this work due to its resistance to oxidation even at elevated temperatures. However, despite these merits noble metals do not tend to exhibit sufficiently strong adhesion to dielectric materials, which is vital for creating robust optical structures that can withstand long-term atmospheric exposure or thermal expansion effects. To mitigate this, ultrathin layers other metals with strong adhesion to both Au and dielectrics, such as Ti, Cr, Ni, can be intercalated. However, these metals exhibit considerably higher ohmic losses and are less reflective, hence, are known to be deleterious to the overall optical performance of a metal based optical structure.

**Figure 6:**
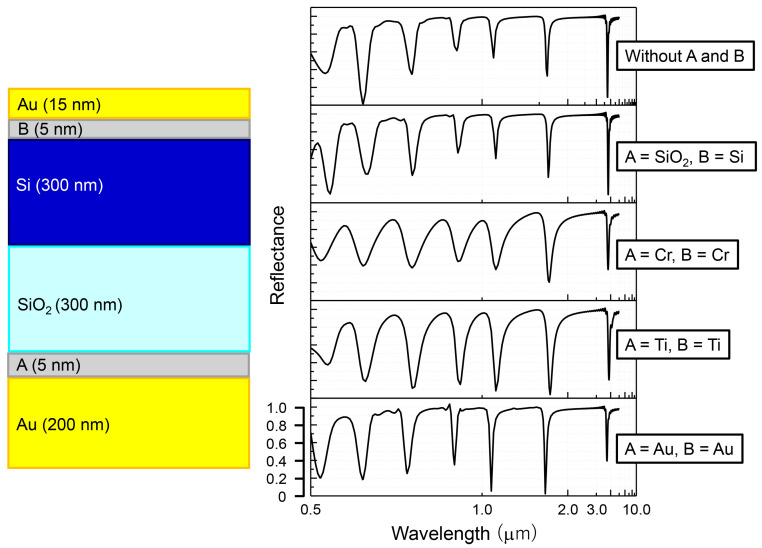
Influence of adhesion layers estimated by way of FDTD simulations. Sketch on the left shows the model structure, where *A* and *B* represent the additional adhesion promoting layers. Plots on the right give the simulated reflectance spectra with various configurations and metals applied for these layers.

Our motivation to use a Ti adhesion promoting layer of minimal thickness can be clarified by considering the FDTD simulation results given in Figure 6. Here for simplicity a single base structure is considered in which both Si and ${\mathrm{SiO}}_{2}$ layers are set to the same 300 nm thickness. The 5 nm thickness layers labeled *A* and *B* are situated where adhesion promoting layers are required. The reference cases are provided by simulated reflectance spectra when these layers are omitted completely, as well as when they match the respective dielectric (*A* = ${\mathrm{SiO}}_{2}$, *B* = Si), or the gold layer (*A* = *B* = Au) to account for variations in total optical path and metal fraction, respectively. All of these reference cases behave in an quite similar manner, with the only notable variance being a slightly increased overall absorbance in the *A* = *B* = Au case due to a higher metal fraction. On the other hand, inclusion of either Ti and Cr adhesion layers possess lower absorbance than in the reference cases as well as a broadening of peaks, attributable to a decreased quality factor of the structure due to higher losses. Cr in particular, in addition to its susceptibility to oxidation, exhibits considerable ohmic loss, hence, resonances in such a case are even broader and anti-reflection performance is diminished as well. For these reasons Ti was chosen as a satisfactory compromise that can provide robust adhesion with acceptable optical performance.

### 3.4. Comparison to the Other Thermal Radiation Materials

**Figure 7:**
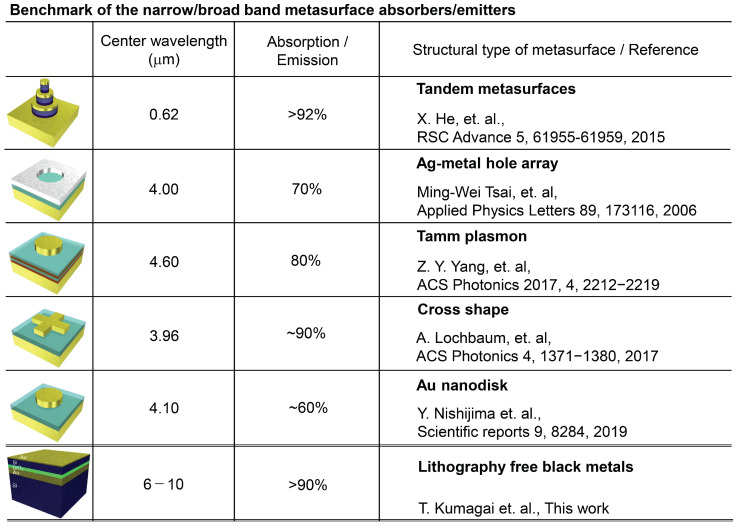
Comparison of the absorption and thermal radiation that reported in the reference articles. He, Tsai, Yang, Lochbaum and our works [2,6,8,29,42].

Among the most widely employed approaches for creating thermal radiation absorber/emitter structures is designing various metasurfaces comprised of a rich variety of noble metal nanoparticle, reflector and dielectric spacer layer configurations. Different thermal emitter metasurfaces reported in the literature are outlined and contrasted with the present work by the summary in Figure 7. In particular, the center wavelength and absorption/emission efficiencies are highlighted as benchmark values. It is evident that in the MIR wavelength range best examples of different microscale patterned metasurfaces converge on an approximately 80–90% absorption/emission efficiency. However, despite being considerably simpler than their lithographic counterparts, black metal absorber/emitter structures exhibit equivalent performance. On the other hand, one expected drawback of a multi-layer structure is that its spectral behavior is markedly more dependent on radiation incidence angle than microstructured variants in which periodic arrangement can fulfill a *k*-vector matching function. However, in many cases operation at a fixed absorbance/emission direction is fully adequate and additional cost and complexity involved in lithographic patterning is not justified. In such cases the simple lithography free black metals have high potential for application as thermal radiation materials.

## 4. Conclusions and Outlook

A simple two-layer Si-${\mathrm{SiO}}_{2}$ structure placed between Au layers acts as an absorber and thermal emitter over the IR-MIR spectral window, which is widely used for molecular fingerprinting in sensor applications and is rapidly gaining importance in emerging radiative cooling approaches. Close to perfect absorber performance was achieved at longer mid-IR wavelength range with emittance also approaching 95% of the black body radiation.

True perfect absorption and thermal radiation emission is still challenging to achieve with metasurfaces despite the considerable cost and technical effort needed to create them. The benefits of black metals, simple 2D surfaces where scattering is small enough and can be ignored, include a performance comparable to that of metasurfaces made out of nanostructures at a fraction of the cost and over large areas. Also, frequency selective thermal radiation simplifies use of wavelength-specific optical filters which are both essential and costly components in thermal radiation sources required for IR gas sensors. Competitive radiation output efficiency and low cost of proposed layered structures, therefore, provide a strong benefit in comparison with currently available filters and materials available for IR sources.

The atmospheric transmission window with T≈90% between 3 and 4 μm wavelengths and slightly lower transmisivity at the 8–13 μm band can be utilised for radiative cooling [43,44] using simple black metal coatings studied in this work. Furthermore, radiation of heat into the 8–13 μm band spectral window where most of organic materials strongly absorb [45,46,47,48], e.g., protein (amide) and DNA/RNA bands common for silk, bacteria and viruses can be harnessed for enhancing biocidal functions of nanotextured materials. The IR emission of hot photo-excited electrons into the IR absorption band of photo-polymerisable resists is shown to contribute to 3D polymerisation when ultra-short laser pulses are used [49]. Another promising area of application is in camouflage [50,51,52], where hiding thermal signatures of objects at the air transparency window while re-radiating heat via black body emission at a different spectral range can benefit from the presented simple multi-layer coatings. In addition, thermo-electrical energy harvesting combined with semiconductor solar cells can benefit from broad band engineering of absorbance and reflectivity [53]. Among more advanced uses, it was demonstrated that the second harmonic generation (SHG) from metasurfaces composed of lithographically defined arrays of plasmonic nanoparticles can be enhanced by phase engineering of reflections in the nanoparticle-silica-Si layers [54]. The strongest SHG was observed at the enhanced reflectivity spectral band. By nanopatterning of the layered coatings proposed in this study at the strong reflection near-IR bands using a direct laser ablation [55] or focused ion beam (FIB) milling [56], a simpler route to fabricating of SHG metasurfaces is an inviting strategy.
